# Cigarette smoking during breastfeeding in Papua New Guinea: Prevalence and demographic and socio-economic predictors

**DOI:** 10.1371/journal.pone.0278373

**Published:** 2022-12-01

**Authors:** Prince Peprah, Williams Agyemang-Duah, Naomi Gyamfi, Bernard Yeboah-Asiamah Asare, Dickson Boateng, Joseph Oduro Appiah, Collins Adu

**Affiliations:** 1 Social Policy Research Centre, University of New South Wales, Sydney, Australia; 2 Centre for Primary Health Care and Equity, University of New South Wales, Sydney, Australia; 3 Department of Geography and Planning, Queen’s University, Kingston, Ontario, Canada; 4 Faculty of Medicine and Health, School of Health, University of New England, Armidale NSW, Australia; 5 Faculty of Health Sciences, Curtin School of Population Health, Curtin University, Kent Street, Perth, Australia; 6 Institute of Applied of Health Sciences, University of Aberdeen, Aberdeen, Scotland, United Kingdom; 7 Department of Geography, Mary Immaculate College, University of Limerick, Limerick, Ireland; 8 University of Northern British Columbia, Prince George, BC, Canada; 9 Department of Health Promotion, Education and Disability Studies, Kwame Nkrumah University of Science and Technology, Kumasi, Ghana; 10 College of Public Health, Medical and Veterniary Sciences, James Cook University, Townsville, Queensland, Australia; University of Zurich, SWITZERLAND

## Abstract

**Background:**

Cigarette smoking during breastfeeding is reported to contribute to significant changes in the composition of breast milk not only by reducing its protective features but also affecting infants’ response to breastfeeding and breast milk. However, studies on the prevalence of cigarette smoking and associated factors during breastfeeding are limited in Papua New Guinea (PNG). This study estimates the prevalence of cigarette smoking and its association with demographic and economic factors among breastfeeding women in PNG.

**Methods:**

We used weighted survey data from the 2016–2018 PNG Demographic and Health Survey (PNGDHS). A weighted sample of 3,822 women who were breastfeeding during the survey were included in the study. The outcome variable in the present study is current cigarette smoking. A multiple logistic regression analysis was used to estimate the association between current cigarette smoking status and socio-demographic and economic variables of breastfeeding women. The regression analysis results were reported using adjusted odds ratios (aOR) with their respective 95% confidence intervals (CIs).

**Results:**

From the weighted sample, the prevalence of cigarette smoking among breastfeeding women was 21.9%; of which 60.8% smoked daily. The mean number of cigarettes smoked in the last 24 hours preceding the survey was 6.05(SD = 5.99). Multiple logistic regression analysis revealed that breastfeeding women who were from the Momase (aOR: 2.337, CI: 1.786–3.058, p<0.001) and Highlands (AOR: 1.589, CI: 1.213–2.082, p = 0.001), had no religious affiliation (aOR: 3.665, CI: 1.235–10.877, p = 0.019), and households with daughters as household heads (aOR: 1.901, CI: 1.231–2.935, p = 0.004) and being in more than one union (aOR: 2.374, CI: 1.805–3.123, p<0.001) were significantly more likely to smoke cigarette compared to women from southern region, those affiliated to Anglican church, those with husband as household heads, and being in one union respectively.

**Conclusion:**

Cigarette smoking among breastfeeding women in PNG is relatively high, and region of residence, religion, relationship to household head, and the number of unions remain independent predictors. Interventions should target the individual socio-economic and cultural contexts within which breastfeeding occurs.

## Background

Cigarette smoking is a public health threat, and regardless of the form it takes, it is very harmful to human health. Smoking leads to diseases such as heart disease, stroke, lung diseases, diabetes, and chronic obstructive pulmonary disease (COPD), which includes emphysema and chronic bronchitis [1]. Smoking also increases the risk for tuberculosis, eye diseases, and problems of the immune system, including rheumatoid arthritis. Thus smoking nearly harms every organ of the body, and consequently kills half of its users,constituting about 8 million people annually [2]. Ten percent of these annual deaths result from passive or secondhand smoking [2]. Globally, cigarette smoking is the dominant form of smoking and is regarded as the gravest lifestyle threat to public health [2,3]. Following its impact on public health,specifically its contribution to lung cancer, there is a global call to end its usage through several initiatives/campaigns such as the celebration of the World No Tobacco Day every year [4].

The prevalence of cigarette smoking has witnessed a marked decline in the western world however, there seems to be a rise in users in low-and-middle-income countries [2,3]. Pierce et al. [3] proposed that a comprehensive framework that involves interventions such as increases in the price of products, mass, smoke-free policies, and restrictions on marketing opportunities would be needed to stop this rise. This strategy has been adopted by countries like the USA and the UK hence, a decline from peak levels of cigarette smoking [3].

The past decade has witnessed an increase in the prevalence of cigarette smoking among women. Consequently, the 2010 World No Tobacco Day had a theme "Gender and tobacco with an emphasis on marketing to women" to combat smoking among women [5]. Maternal smoking is the single most common preventable cause of stillbirth and adverse effects on pregnancy [6]. Smoking during pregnancy can damage the tissues (e.g lung and brain) of an unborn baby and can cause cleft lip among children [7,8]. Although pregnant women are counselled not to smoke, the highly addictive nature of smoking makes it almost an impossible act for many pregnant women [2,7,8]. Previous studies have reported that even in post-delivery, some women continue to smoke cigarettes and hence during breastfeeding period [9,10]. Meanwhile, many studies have shown the importance of breastmilk for newborns [11–13]. Thus women are encouraged to breastfeed their children even if they do not stop smoking [14]. Like the pregnancy periods, smoking while breastfeeding affects the health of the newborn. It is known to contribute to a reduction in the iodine content of milk which leads to unhealthy reactions by the newborn [14,15]. It has also been documented that mothers’ smoking during breastfeeding distorts the sleeping patterns of newborns [14]. Furthermore, women who smoke are less likely to breastfeed [16]; such women tend to think that their milk is inadequate and hence, unlikely to breastfeed their newborns [17,18]. Again, women who smoke are more likely to wean their babies earlier than those who did not smoke. Thus, smoking during breastfeeding tends to have a direct effect on mothers which in the long run, affects their newborns. Generally, some studies have reported an association between demographic and socio-economic predictors of women and cigarette smoking [19,20]. Women who are less educated, living in rented accommodation, single, and having a partner who smokes tend to smoke during pregnancy [19]. A study restricted to lone mothers, Siahpush et al. [20] found that less-educated women, younger women, women who lived in disadvantaged areas, women who received government benefits and lived in rented accommodation are more likely to smoke. Despite this, studies that investigate the association between demographic and socio-economic predictors of cigarette smoking among breastfeeding women especially in low-and-middle-income country settings are limited.Cigarette smoking is the only risk factor that is shared by all four major noncommunicable diseases (NCDs) cancer, cardiovascular disease, diabetes, and respiratory disease and it is also the most avoidable cause of NCDs [19].

Generally, studies on cigarette smoking among breastfeeding women tend to be ‘western-focused’ despite the problem persisting also in the Global South [21–23]. In Papua New Guinea (PNG) for instance, the few studies on cigarette smoking have mostly focused on the status of users and prevention [24–26] where it was ascertained that there was a high incidence of smoking. Despite the country ratifying the WHO Framework Convention on Tobacco Control and passing the Tobacco Act, the Tobacco Products (Health Control) Act 1987, there persists a high smoking incidence [26]. Every year, 12,800 people in PNG are predicted to die from diseases linked to cigarette smoking [26]. Despite this, roughly 17% of women continue to smoke daily [26]. To the best of our knowledge, no study on cigarette smoking during breastfeeding among women has been conducted in PNG. This present study therefore examines the prevalence, demographic and socio-economic predictors of cigarette smoking during breastfeeding among women using data from the 2016–2018 PNG Demography and Health Survey. Findings from this study will build on public health studies on cigarette smoking and further inform policy formulation in PNG and other developing countries sharing similar demographic, socio-economic and health characteristics with PNG.

## Materials and methods

### Sample

The study used weighted survey data from the 2016–2018 PNG Demography and Health Survey (PNGDHS) conducted from October 2016 to December 2018. The PNG DHS aimed at generating comprehensive data on demographic, maternal and reproductive issues such as fertility, family planning awareness and practices, breastfeeding practices, health behaviours, immunizations, and domestic and intimate partner violence. Through the Demographic and Health Survey (DHS) program, technical support for the execution of the survey was provided by Inner City Fund (ICF), with the financial support of the PNG Government, Australian Government Department of Foreign Affairs and Trade, the United Nations Population Fund (UNFPA) and UNICEF [27]. The sample for the 2016–18 PNG DHS was nationally representative and covered the entire population that lived in private dwelling units in the country. The survey used the list of census units (CUs) from the 2011 PNG National Population and Housing Census as the sampling frame and adopted a probability-based sampling approach.

Specifically, a two-stage stratified cluster sampling procedure was followed. Details of the methodology and selection procedure have been reported in the PNG DHS final report (see https://dhsprogram.com/publications/publication-fr364-dhs-final-reports.cfm). In summary, each province in the country was stratified into urban and rural areas, yielding 43 sampling strata, except the National Capital District, which has no rural areas. The division paid particular attention to urban-rural variations. Samples of census units were selected independently in each stratum in two stages. In the first stage, sorting of the sampling frame within each sampling stratum to achieve implicit stratification and proportional allocation using a probability proportional-to-size selection was done. In the second stage of sampling, a fixed number of 24 households per cluster were selected with an equal probability systematic selection from the newly created household listing, resulting in a total sample size of approximately 19,200 households. To prevent bias, no replacements and no changes of the pre-selected households were allowed in the implementing stages. In cases in which a census unit had fewer than 24 households, all households were included in the sample.

A total of 17,505 households were selected for the sample, of which 16,754 were occupied. Of the occupied households, 16,021 were successfully interviewed, yielding a response rate of 96%. In the interviewed households, 18,175 women aged 15–49 were identified for individual interviews; interviews were completed with 15,198 women, yielding a response rate of 84%. In the subsample of households selected for the male survey, 9,141 men aged 15–49 were identified and 7,333 were successfully interviewed, yielding a response rate of 80%. In this present study, the weighted sample comprised 3,822 women who were currently breastfeeding during the survey. Thus, our analysis used data only on women who were breastfeeding during the survey. The dataset can be accessed at https://dhsprogram.com/data/dataset/Papua-New-Guinea_Standard-DHS_2017.cfm?flag=0.

### Variables

#### Dependent variable

Breastfeeding women provided “no” (0) or “yes” (1) responses to a single item: whether they had smoked in the last 24 hours prior to the survey. Thus, breastfeeding women’s current cigarette smoking status was considered to be the outcome variable in this study.

#### Independent variables

We included theoretically and empirically relevant demographic and socioeconomic variables as explanatory variables. In all, we included eight (8) explanatory variables including the region of residence, religion, relationship to household head, internet usage, wealth status, number of unions, current marital status and residing with a partner based on their significance from the chi-square analysis and availability in the datasets as well as evidence from previous studies [28–31]. We did not recode any of these variables. Region was grouped into four categories: Southern, Highlands, Momase and Islands regions. Religious status was classified into 12 categories: Anglican, Evangelical Alliance, Pentecostal, Evangelical Lutheran, Roman Catholic, Salvation Army, Seventh Day Adventist, United Church, Other Christian Church, Non-Christian and No religion. Relationship to household head was grouped as head (husband refers to the respondent being the head of the household), wife, daughter, daughter-in-law, grandparent, sister, other relatives, adopted/foster child and not related. Internet usage was categorized as never; yes, last 12 months; and yes, before last 12 months. Wealth status was categorized as poorest, poorer, middle, richer and richest. The number of intimate unions (marriage or cohabitation) was captured as: once and more than once while current marital status was classified into married and living with a partner. Lastly, currently residing with a husband/partner was captured as living with him and staying elsewhere (Table 1).

**Table 1:** Demographic and socio-economic characteristics of the participants by cigarette smoking status (N = 3822).

Variables	Cigarette smoking status						
	Weighted N No		Yes		Total		p-value
	Weighted	%	Weighted N	Weighted %	Weighted N	Weighted %	
**Region**							
Southern region	925	31.0	174	20.8%	1099	28.8	
Highlands region	744	24.9	218	26.0	962	25.2	
Momase region	596	20.0	276	33.0	872	22.8	<0.001*
Islands region	720	24.1	169	20.2	889	23.3	
**Highest educational level**							
No education	656	22.0	216	25.8	872	22.8	
Primary	1545	51.8	397	47.4	1942	50.8	0.083
Secondary	702	23.5	200	23.9	902	23.6	
Higher	82	2.7	24	2.9	106	2.8	
**Current marital status**							
Never in union	94	3.1	33	3.9	127	3.3	
Married	2282	76.4	605	72.3	2887	75.5	
Living with partner	470	15.7	139	16.6	609	15.9	
Widowed	19	0.6	10	1.2	29	0.8	
Divorced	16	0.5	5	0.6	21	0.5	
No longer living together/separated	104	3.5	45	5.4	149	3.9	0.038*
**Number of unions**							
Once	2662	92.8	674	85.1	3336	91.1	
More than once	206	7.2	118	14.9	324	8.9	<0.001*
**Currently residing with husband/partner**							
Living with him	2378	86.7	611	82.9	2989	85.9	
Staying elsewhere	366	13.3	126	17.1	492	14.1	0.009*
**Religion**							
Anglican	116	3.9	32	3.8	148	3.9	
Evangelical Alliance	78	2.6	22	2.6	100	2.6	
Pentecostal	248	8.3	68	8.1	316	8.3	
Evangelical Lutheran	271	9.1	111	13.3	382	10.0	<0.001*
Roman Catholic	778	26.1	292	35.0	1070	28.1	
Salvation Army	9	0.3	1	0.1	10	0.3	
Seventh Day Adventist	407	13.7	79	9.5	486	12.7	
United Church	410	13.8	71	8.5	481	12.6	
Other Christian Church	641	21.5	142	17.0	783	20.5	
Non-Christian	13	0.4	7	0.8	20	0.5	
No religion	7	0.2	10	1.2	17	0.4	
**Relationship to household head**							
Husband	224	7.5	61	7.3	285	7.5	
Wife	1951	65.4	490	58.5	2441	63.9	
Daughter	416	13.9	149	17.8	565	14.8	
Daughter-in-law	173	5.8	58	6.9	231	6.0	
Granddaughter	10	0.3	6	0.7	16	0.4	
Sister	53	1.8	24	2.9	77	2.0	0.007*,
Other relative	116	3.9	39	4.7	155	4.1	
Adopted/foster child	18	0.6	6	0.7	24	0.6	
Not related	24	0.8	4	0.5	28	0.7	
**Sex of household head**							
Male	2576	86.3	711	84.9	3287	86.0	0.319
Female	409	13.7	126	15.1	535	14.0	
**Literacy**							
Cannot read at all	1006	33.9	319	38.5	1325	34.9	
Able to read only parts of sentence	550	18.5	135	16.3	685	18.0	
Able to read whole sentence	1381	46.5	369	44.5	1750	46.1	
No card with required language	28	0.9	6	0.7	34	0.9	0.126
Blind/visually impaired	2	0.1	0	0.0	2	0.1	
**Owns a mobile telephone**							
No	2136	71.8	586	70.6	2722	71.5	
Yes	841	28.2	244	29.4	1085	28.5	0.517
**Use mobile phone for financial transactions**							
No	679	81.6	208	86.0	887	82.6	
Yes	153	18.4	34	14.0	187	17.4	0.117
**Own a financial account**							
No	2478	83.6	686	83.0	3164	83.5	0.641
Yes	485	16.4	141	17.0	626	16.5	
**Use of internet**							
Never	2739	92.1	731	88.0	3470	91.2	
Yes, last 12 months	175	5.9	81	9.7	256	6.7	
Yes, before last 12 months	59	2.0	19	2.3	78	2.1	<0.001*
**Wealth index**							
Poorest	551	18.5	156	18.6	707	18.5	
Poorer	507	17.0	143	17.1	650	17.0	0.034*
Middle	602	20.2	140	16.7	742	19.4	
Richer	725	24.3	193	23.1	918	24.0	
Richest	600	20.1	205	24.5	805	21.1	
**Covered by health insurance**							
No	2892	97.2	811	97.0	3703	97.1	
Yes	84	2.8	25	3.0	109	2.9	0.797
**Age in 5-year groups**							
15–19	156	5.2	43	5.1	199	5.2	
20–24	687	23.0	204	24.4	891	23.3	0.344
25–29	788	26.4	233	27.8	1021	26.7	
30–34	624	20.9	184	22.0	808	21.1	
35–39	478	16.0	107	12.8	585	15.3	
40–44	192	6.4	47	5.6	239	6.3	
45–49	60	2.0	19	2.3	79	2.1	
**Currently working**							
No	2031	68.2	569	68.1	2600	68.2	0.965
Yes	946	31.8	266	31.9	1212	31.8	

* indicate significance of *p* value (*p* < 0.05).

### Statistical analysis

Data analysis was conducted using SPSS software v.20 (IBM, Armonk, NY) and followed three main steps. First, we performed descriptive statistics such as frequencies, percentages, mean, and standard deviation to describe the background characteristics of the study participants and establish the prevalence of cigarette smoking among the sample. Second, a bivariate analysis was performed using chi-square (χ^2^) to identify and select suitable variables for the regression analysis. Significant variables with a p-value of 0.05 or less were selected for the multiple logistic regression analysis. Before the regression analysis, diagnostics checks for multicollinearity were conducted using the variance inflation factor (VIF). In this analysis, none of the VIF scores exceeded the value of 2.38, suggesting no multicollinearity. In the final stage, a multiple logistic regression was performed to determine the odds of cigarette smoking in relation to demographic and socio-economic variables during breastfeeding. The regression analysis output was reported as adjusted odds ratios (aORs) with their corresponding 95% confidence intervals at a p-value .05 or less as significant. The PNGDHS provided appropriate sampling weights for this study, which were applied to derive all prevalence estimates. Thus, all the analyses were done accounting for the complex survey design in SPSS.

## Results

### Demographic and socio-economic characteristics of the participants by cigarette smoking status

Of 3,822 participants, 28.8% were from the southern region of Papua New Guinea, 50.8% had attained primary education, 46.1% were able to read a full sentence, 75.5% were currently married, 85.9% of the married women were currently residing with their husbands/partners, and 26.7% were aged 25–29 years (see Table 1). The study found that 28.1% of the participants were Roman Catholic while 86% of the household heads were males. Approximately 17% of the participants had an account in a bank or other financial institution. Slightly above 91% of the participants had never used the internet in their lifetime. Concerning the employment status, 31.8% of the participants were working and 24% of them rated themselves as ‘richer’. The study had revealed that 2. 9% of the participants were covered in a national health insurance scheme (see Table 1).

### Prevalence of cigarette smoking among breastfeeding women

Of weighted sample of 3,822 breastfeeding women, 21.9% were smoking cigarettes. Of the 21.9% of the participants who were smoking cigarettes, 60.8% of them smoked cigarettes daily. The mean number of cigarettes smoked (± SD) in the last 24 hours preceding the survey of the overall sample was 6.05 (5.994) (see Table 2).

**Table 2:** Prevalence and patterns of cigarette smoking (N = 3822).

Variable	Weighted N	Weighted %	Mean(SD)
**Smokes cigarette**			-
No	2985	78.1	
Yes	837	21.9	
**Frequency of cigarette smoking**			
Every day	509	60.8	-
Some days	328	39.2	-
**Number of cigarette in last 24 hours**	-	-	6.05(5.99)

### Demographic and socio-economic factors associated with cigarette smoking in breastfeeding

The chi-square analysis showed significant differences between region (p = 0.000), current marital status (p = .038), number of intimate unions (p<0.001), residing with a husband/partner (p = 0.009), religion (p<0.001), relationship to household head (p = 0.007), use of internet (p<0.001) and wealth index (p = 0.034) and cigarette smoking (see Table 1).

Multiple logistic regression revealed that region of residence, religion, relationship to household head, and the number of unions were independent predictors of cigarette smoking during breastfeeding (Table 3). The results show that compared with breastfeeding women from the Southern region, coming from Momase (aOR: 2.337, CI: 1.786–3.058, p<0.001) and highlands (aOR: 1.589, CI: 1.213–2.082, p = .001) regions significantly had higher odds of cigarette smoking during breastfeeding. Breastfeeding women with no religious affiliation (aOR: 3.665, CI: 1.235–10.877, p = 0.019) had higher odds of cigarette smoking during breastfeeding compared to those who were affiliated with the Anglican Church. On the contrary, breastfeeding women who were Seventh Day Adventists (AOR: 0.496, CI: 0.296–0.830, p = .008), United Church (aOR: 0.540, CI: 0.323-.903, p = 0.019) and other Christian churches (aOR: 0.566, CI: 0.348–0.919, p = 0.021) significantly lower odds of smoking cigarettes during breastfeeding compared to those who were Anglicans. Further, relationship to household head was associated with cigarette smoking during breastfeeding such that having a household head as a daughter (aOR: 1.901, CI: 1.231–2.935, p = 0.004) significantly had higher odds of smoking cigarettes during breastfeeding compared to those who responded that they were head of their households. In addition, being in an intimate union more than once (aOR: 2.374, CI: 1.805–3.123, p<0.001) significantly had higher odds of smoking cigarettes during breastfeeding compared to those who had been in an intimate union for once (see Table 3).

**Table 3:** Multiple logistic regression analysis on factors associated with cigarette smoking.

Variables	AOR	95% CI		p-value
		Lower	Upper	
	**Region**				
	Southern region (ref)	1.00			
	Highlands region	1.589	1.213	2.082	0.001*
	Momase region	2.337	1.786	3.058	<0.001*
	Islands region	1.036	0.783	1.371	0.803
	**Religion**				
	Anglican (ref)	1.00			
	Evangelical Alliance	0.759	0.382	1.509	0.431
	Pentecostal	0.624	0.365	1.067	0.085
	Evangelical Lutheran	0.858	0.512	1.437	0.560
	Roman Catholic	1.111	0.694	1.778	0.662
	Seventh Day Adventist	0.496	0.296	0.830	0.008*
	United Church	0.540	0.323	0.903	0.019*
	Other Christian Church	0.566	0.348	0.919	0.021*
	Non-Christian	2.061	0.655	6.478	0.216
	No religion	3.665	1.235	10.877	0.019*
	**Relationship to household head**				
	Husband (ref)	1.00			
	Wife	1.326	0.874	2.011	0.185
	Daughter	1.901	1.231	2.935	0.004*
	Daughter-in-law	1.881	1.135	3.119	0.014*
	Granddaughter	3.969	1.021	15.434	0.047*
	Sister	1.884	0.951	3.735	0.070
	Other relative	1.593	0.902	2.814	0.109
	Adopted/foster child	1.183	0.301	4.650	0.810
	Not related	0.438	0.095	2.008	0.288
	**Use of Internet**				
	Never (ref)	1.00			
	Yes, last 12 months	1.373	0.977	1.930	0.068
	Yes, before last 12 months	0.948	0.512	1.757	0.866
	**Wealth index**				
	Poorest (ref)	1.00			
	Poorer	0.992	0.751	1.312	0.958
	Middle	0.787	0.591	1.049	0.102
	Richer	1.003	0.756	1.330	0.985
	Richest	1.210	0.899	1.627	0.209
	**Number of Unions**				
	Once (ref)	1.00			
	More than once	2.374	1.805	3.123	<0.001*
	**Currently residing with a husband/partner**				
	Living with him (ref)	1.00			
	Staying elsewhere	1.226	0.912	1.649	0.178
	**Current marital status**				
	Married (ref)	1.00			
	Living with partner	.984	.786	1.230	0.885
	**Model fitting information**				
	Omnibus Tests of Model Coefficients (sig)				209.411 (0.000)
	Hosmer and Lemeshow Test (sig)				9.525 (0.300)
	Estimate with correct classification (%)				79.0
	-2 Log likelihood				3319.601
	Cox & Snell R Square				0.059
	Nagelkerke R Square				0.092

*indicate significance of *p* value (*p* < 0.05).

AOR = Adjusted Odd Ratio; CI = Confidence Interval; Ref = Reference Group.

## Discussion

The present study was aimed at examining the prevalence and demographic and socio-economic predictors of cigarette smoking among breastfeeding women in PNG. The prevalence of cigarette smoking during breastfeeding was 21.9%, of which 60.8% smoked daily. The study further found statistically significant association between cigarette smoking and demographic factors including region of residence, religion, relationship to household head, and the number of unions among breastfeeding women in PNG.

The prevalence of cigarette smoking among breastfeeding women in the present study is comparable to the finding of a study conducted in Italy [32], but higher than in a study conducted in Canada [9]. Additionally, the prevalence of cigarette smoking among breastfeeding women in the present study was lower than in a study conducted in Turkey [33]. The reasons for the observed difference may include socio-cultural differences and the methodological approaches adopted for the various studies in these countries.

For low- and middle-income countries data on cigarette smoking during breastfeeding are limited. Although our analysis did not include the health implications of cigarette smoking on breastfeeding, some previous studies have highlighted the consequences of cigarette smoking during breastfeeding [14,21,34]. For instance, cigarette smoking among breastfeeding women is said to contribute to infant health problems [21,34]. Smoking may adversely affect the nutritional composition of breast milk and can also decrease a new mother’s breastmilk supply, shortening lactation duration [21]. Contaminated breastmilk especially by nicotine, a harmful chemical secreted into breastmilk can cause a reduction in iodine supply, changes in sleep and wakefulness patterns [14,35] damage on liver and lung; reduction of pancreatic β cells; intracellular oxidative damage; and decreased glucose tolerance among babies [35].

Studies on the predictors of cigarette smoking during breastfeeding are rare though some studies have focused on experiences of breastfeeding women living with people who smoke [36–38] and depression [38–40]. In this present study, region of residence was an independent predictor of cigarette smoking during breastfeeding. The results showed that compared with breastfeeding women from the Southern region, coming from Momase and Highlands regions significantly had higher odds of cigarette smoking during breastfeeding. Similar to finding of a previous study among the general population in PNG where people from the Momase, Highlands and Islands compared to Southern region were more likely to consume tobacco [41]. Disparities in smoking levels among pregnancy women have also been documented across the provinces and territories in Canada [42]. PNG has a diverse demographic background [43], as such have diverse ethnic and cultural practices, educational levels and socio-economic status across the different regions. As such, there may be disparities in geographical access to and distribution of cigarette (or tobacco products) [41]. Again, there may be differences in the access to health and health information or campaign such as awareness creation and education on the health risks of cigarette smoking on breastfeeding women and their newborns across the difference regions. These have the potential to explain the disparities in the likelihood of cigarette smoking across the geographical regions. Efforts should be made to provide intensive awareness creation and health education to women in these regions, highlighting the negative consequences of smoking during pregnancy and breastfeeding; possibly through mass campaigns and during prenatal and postnatal visits.

Consistent with the findings of other studies [44,45], religion was found an independent predictor of cigarette smoking during breastfeeding. Correspondingly, those who had no religion were more likely to smoke as compared to those who had religious affiliations. Religious affiliation has been identified as a protective factor, it offers social support that may minimize the social stresses encountered by women [46]. Furthermore, people who are more affiliated to a particular religious denomination may perceive that smoking cigarette is against their religion/beliefs, as most religion do not approve of tobacco use [47]. Also, people who are affiliated to a specific religious orientation may have the obligation of setting a good lifestyle and/or living exemplary lives to attract others to God and as such, are less likely to smoke cigarette. Therefore, interventions on smoking cessation among breastfeeding women can be targeted and geared towards religious domains. Public health advocates could join with religious bodies to advance knowledge in this area to stop cigarette smoking among breastfeeding women.

Further, the relationship to household head influenced cigarette smoking during breastfeeding such that having a household head as a daughter significantly had higher odds of smoking cigarettes during breastfeeding. Smoking has been indicated to be associated with the poor authority or supervision by the head of family or household due to the lack of mental and physical capabilities [48]. Societal norms that require children (young or old) to be respectful and obedient to their parents and see the elderly of knowing what is good, could suggest daughters as the head of households could lack the moral power to exert the authority to supervise their mothers to stop engaging in risky behaviours such as smoking. Our finding suggests the need for heads of households particularly those of child to parent relations to be encouraged and supported to develop their mental abilities and the moral strength to have an independent right to advise and encourage new mothers to quit smoking.

Additionally, being in more than one intimate union was significantly associated with smoking cigarettes during breastfeeding. Being in more than one intimate union could suggest one could have experienced losing a partner through death, divorce or separation. Higher levels of smoking have been identified among women who have experienced divorce or marital separation [49,50]. Divorce or marital separation have been indicated to be a significant cause of psychological distress [51], which have been documented to be associated with smoking [38,52] and smoking as a means for coping with stress among women [36,53]. The findings of this study suggest that women who have been in several unions should be identified and offered the necessary support in acquiring the necessary abilities to cope with possible factors that induce their cigarette smoking during breastfeeding smoking. The negative repercussions of cigarette smoking especially during breastfeeding should be emphasized to this group of women.

Our results indicate that cigarette smoking during breastfeeding is quite common in PNG and considering the negative effect of smoking on the babies and their mothers, the present study findings call for interventions targeting maternal smoking prevention in PNG.

### Public health policy, practice and research implications

This study offers possible public health policy, practice, and research implications that need to be commented on. In terms of public health practice, public health interventions in form of education, and sensitization should be organized regularly for breastfeeding mothers who are involved in cigarette smoking in PNG and other developing countries that share similar characteristics with PNG. It is important to state that although the study did not cover the health implications of smoking among breastfeeding mothers in PNG, excessive and frequent smoking of cigarettes could have implications on the milk composition, quality of the breastfeeding as well as the health of the breastfeeding mothers and their children. Thus far, the health sector should be a key stakeholder in the campaign against cigarette smoking during breastfeeding. Specifically, the PNG National Department of Health and other health institutions should lead the campaign against cigarette smoking in breastfeeding. The campaign should cover the potential effects of cigarette smoking on breast milk composition and quality, health risks (chronic diseases, physical health, emotional health and psychosocial health) of cigarette smoking during breastfeeding on both the mothers who smoked and their children (both born and unborn), and the economic and social effects of cigarette smoking.

Concerning the public health policy implications, the findings of our study suggest that a number of factors such as region of residence, religion, relationship to household head, and the number of unions were significant predictors of cigarette smoking during breastfeeding. Specifically, any new program or policy that seeks to scale down cigarette smoking during breastfeeding should consider the above significant factors associated with cigarette smoking. For instance, the findings further suggest that since breastfeeding women coming from Momase and highlands regions were more likely to smoke cigarettes, the PNG health institutions should limit access to cigarettes for women residing in these areas during breastfeeding. This in a way would help to ensure a reduced likelihood of cigarette smoking among breastfeeding women from Momase and highlands regions of PNG. In addition, PNG health institutions and other health organisations should provide smoking cessation programmes, including as behaviour therapy and counselling, to assist women who are breastfeeding in quitting smoking. Cessation programmes for breastfeeding mothers should be held on a regular basis to raise awareness of the negative effects of cigarette smoking on their health and the health of their babies.

In terms of research implications, future research should consider the knowledge and awareness and associated factors of health risks of cigarette smoking during breastfeeding on mothers and their children in PNG. Also, such study should consider how women who smoked cigarettes during breastfeeding consider their health status and that of their children in terms of chronic diseases, physical, emotional, psycho-social health. In addition, since this study did not look at the various health factors (in terms of chronic diseases, physical, emotional, psycho-social health) associated with cigarette smoking among women who smoked during breastfeeding, future study should delve into these important research areas to inform policy decision in relation to cigarette smoking during breastfeeding. Lastly, it is also important that further research should investigate the motivations for cigarette smoking during breastfeeding.

### Strengths and weaknesses

The strength of the study is that to the best of our knowledge, it is the first study that analyzes data from a nationally representative sample regarding the prevalence and demographic and socio-economic correlates of cigarette smoking during breastfeeding in PNG. Though the study findings can be generalized, they are limited to some extent thus our findings should be viewed and interpreted in the context of the following limitations The study relied on cross-sectional data and as such causal interpretations of the findings are limited to an extent. The study relied on data collected through self-reporting which could not be independently verified, and as such their prevalence of cigarette smoking and demographic as well as socio-economic details such as wealth index could be under-or over-estimated. In addition to being limited to self-report, the measure of cigarette smoking is limited to a dichotomous indication of smoking only in the past 24 hours. Thus, women who smoke occasionally during breastfeeding may have been missed because they smoked outside of that 24-hour window. In addition, there is no data on amount of cigarette smoking. For instance, heavier smokers likely will have different demographic and/or socioeconomic indicators than lighter smokers. The study is also limited to the prevalence of cigarette smoking and associated factors and did not explain motivations for smoking during breastfeeding. Again, our analysis did not include the health implications of cigarette smoking on breastfeeding. Thus far, the weaknesses in the present study call for both longitudinal and qualitative studies. Future studies should therefore consider the gaps outlined in the present study.

### Conclusion

The present findings indicate that cigarette smoking is quite common among breastfeeding women in PNG. The study revealed that several demographic and socio-economic characteristics place breastfeeding women at increased risk for cigarette smoking. While region of residence, relationship to household head, and the number of unions increased the risk of cigarette smoking among breastfeeding women, religious affiliation reduces the odds of cigarette. Since religious affiliation decreased the risks of cigarette smoking, health institutions in PNG should actively involve religious institutions in their quest to reduce cigarette smoking among breastfeeding women. In a broader picture, interventional efforts on smoking cessation among breastfeeding women in PNG should take into consideration these predictive factors.
